# Does A Multiple-Sport Intervention Based on the TGfU Pedagogical Model for Physical Education Increase Physical Fitness in Primary School Children?

**DOI:** 10.3390/ijerph17155532

**Published:** 2020-07-31

**Authors:** Armando Cocca, Jovanny Edmundo Carbajal Baca, Germán Hernández Cruz, Michaela Cocca

**Affiliations:** 1 Department of Sport Science, University of Innsbruck, Fürstenweg 185, 6020 Innsbruck, Austria; 2 Degree in Sport Training, State University of Sonora, Boulevard Rosales 189, Colonia Centro, 83079 Hermosillo, Mexico; jovannyed@hotmail.com; 3 School of Sport Organization, Autonomous University of Nuevo Leon, Ciudad Universitaria s/n, 66455 San Nicolás de los Garza, Mexico; cruz_hg@hotmail.com (G.H.C.); michaela.cocca@gmail.com (M.C.)

**Keywords:** physical education, children, physical fitness, pedagogical models, health, sports

## Abstract

Teaching Games for Understanding (TGfU) is one of the pedagogical models used for increasing health through physical education (PE), being associated with several psychological benefits. However, only few studies have studied the effect of TGfU on physical fitness. This study aims at assessing the changes in students’ physical fitness after a six-month TGfU-based program with primary school children. A total of eight schools from the state of Sonora (Mexico) were randomly distributed into experimental (EG) and control group (CG). The final sample consisted of 188 pupils (100 boys, 88 girls; age = 10.22 ± 0.76 years) from the 5th and 6th grade. Employing a quasi-experimental design, physical fitness was assessed by means of the Eurofit test battery. At post-test, EG obtained significantly higher scores than CG in flexibility, abdominals, speed (*p* < 0.001), handgrip (*p* = 0.002), low-limb power (*p* = 0.032), and cardiorespiratory fitness (*p* = 0.048). Our findings suggest that TGfU can be a valid alternative to traditional methodologies not only when the aim of a PE unit is to stimulate the cognitive domain, but also for the development of physical fitness attributes that may help pupils develop in a comprehensive manner.

## 1. Introduction

The potential of schools as main vehicles for improving youth’s health has become more and more evident over the last decades, mostly due to the fact that children and adolescents, regardless of the world region, spend a large portion of their daytime in such environment [1]. However, the advantages of working with schools compared to other social environments are not limited to the mere number of hours youngsters spend in them. Indeed, schools are generally considered safe settings, where kids can learn and develop all the spheres of life with no or low risk [2]. Moreover, most schools offer different services at lower rates (or, when included in the educational curriculum, without charge) than the costs associated with afternoon activities provided by private entities [3]. Furthermore, other barriers at times perceived as insurmountable for kids’ afternoon activities (parents’ time availability, closeness and accessibility of infrastructures, availability of means of transportation, etc.) are less impactful in schools [4]. It is clear that the promotion of health in such a favorable environment can have significant effects not only on the short term, but also on the long, given the strong connection between youth’s development and their future physical, psychological, and social condition as adults [5]. Despite its recognized value and advantages, not all school programs targeting health have shown to be effective. For instance, previous literature highlights how programs not founded on strong evidence may have a negative impact on students’ cognitive and physical assets [6]. A review of physical education interventions showed that only half of the studies obtained significantly positive results [7]. The authors underline how lack of proper and accurate assessment and process evaluation, focus on students’ knowledge over other components that influence health, or the use of designs with low control on external influences may hinder the final outcomes of those programs. On the other hand, other programs made use of protocols that complemented in-school with out-of-school interventions [8]. Consequently, their outcomes cannot be fully justified with the positive impact of the school environment.

In order to trigger health benefits within schools, researchers and practitioners have implemented various strategies aiming at increasing the different domains of health. These strategies have included dietary programs [9], mental health literacy [10], social inclusion and equity [11], or stress reduction [12] among others. Although a variety of areas have been investigated, a considerable body of research and practice focuses on in-school physical activity (PA), due to the essential role played by an active lifestyle in building healthy behaviors at early ages [13]. In-school PA programs cover an array of objectives within the larger area of health, including the enhancement of biological markers [14], the promotion of social behaviors [15], the improvement of the mental domain [16], or the increase of physical fitness [17]. Concurrently with the development of these programs, several authors have proposed innovative pedagogical models created with the aim of facilitating and maximizing their effects within physical education (PE) and, more generally, the school context [18]. Among these models, Teaching Games for Understanding (TGfU; [19]) has become popular over the years as a pedagogical approach for PE thanks to its impact on several youth’s developmental cornerstones that any PE curriculum pursues, such as decision making, enjoyment, or physical activity levels [20]. TGfU is a model created for PE teachers, offering students the possibility of playing modified versions of any sport in such manner that they can learn different aspects of a sports discipline altogether, emphasizing the “why” (tactical meaning of technical executions) certain actions are executed over the “how” (learning to perform a technique by repeating it in isolation) [21,22]. Through this model, pupils develop a deep understanding of sports, not limited to the practical skills associated with performance, but also in terms of tactical awareness or in-game decision making, which are competences that can be transferred into real life [23]. The basic structure of TGfU is founded on six phases [20]: (a) Game presentation, involving discussions and brainstorming on the dynamics of the chosen sport/s; (b) game appreciation, with students practicing the chosen sport/s in smaller groups; (c) tactical awareness, which proposes students’ debates on the tactical elements of the game needed for success; (d) decision making, the core phase of the model, during which students face different in-game situations and try to respond to them with proper decision making and actions; (e) skill execution, focused on training the proper skills needed to positively resolve the situations presented in the previous phase; and (f) performance, which allows students to merge the learnings from the previous phases and perform them simultaneously in simple and complex game situations. Due to the complexity of the teaching-learning processes in sports and the multifaceted nature of this model, the application of TGfU attains its highest value within the PE classroom rather than during other in-school periods [24]. The body of research on the implementation of TGfU in PE is extensive. TGfU-based interventions carried out in different PE settings have shown the model’s positive impact on tactical awareness [25], decision making, enjoyment, and intention to be active [26], motivation and perceived competence [23], academic performance and literacy [27], movement skills [28], or PA levels [29]. Despite its widespread use in school PE, and although several studies have suggested that TGfU may not only trigger a significant development of the cognitive domain but also stimulate moderate-to-vigorous PA levels and higher motivation towards active lifestyles, the assessment of how TGfU school units influence physical fitness remains only marginally explored [30]. Considering the importance of physical fitness towards the proper growth and health status of any individual especially at early ages [31,32,33], the lack of research on the link between TGfU and fitness represents a gap in the literature that should be looked into. In fact, if this model was shown valuable for physical fitness increase, its practical application could expand from a predominantly positive-sport-teaching strategy to more areas of the PE curriculum covering health promotion. For this reason, the objective of this study is to assess the impact of a TGfU unit focused on different sports on physical fitness in primary education students from fifth and sixth grade.

## 2. Materials and Methods

### 2.1. Study Design

Randomization of participants into the experimental and control treatment was not possible due to the presence of pre-existing structured groups within the schools involved in the study. As preserving these groups was required, the study was built on a quasi-experimental pre-post design [34]. The independent variable was PA through PE with two levels of application, the first being an intervention program based on TGfU, the second the traditional PE curriculum for sports. Speed, flexibility, cardiorespiratory fitness, and muscular strength, power, and endurance constituted the dependent variables.

### 2.2. Participants

Primary schools in the urban area of Benito Juarez, Sonora (Mexico) were considered as the initial universe for our study. Primary education is an essential stage, as children build up the physical, cognitive, and social foundations which will later contribute to their proper development [35]. In this sense, primary education PE is regarded as a critical discipline that may bring benefits in all areas of an individual’s development [36]. A total of eight primary schools were included in the study. School selection was by convenience and based on directors’ permission, i.e., only those schools whose directors allowed the research to be carried out at their facilities. All schools presented a similar socioeconomic condition, comparable facilities, and were located in neighborhoods within the city skirt. Treating the schools as conglomerates, the initial process of sample selection involved a randomized assignment of each school to either experimental (EG, four schools), or control group (CG, four schools). Within each school, the inclusion criteria for the students to be eligible were: (a) Being enrolled in either 5th or 6th grade: We decided to narrow the study to kids of 10 years of age or older due to the complexity of the model and the higher order thinking skills required to carry it out effectively [22]; (b) not exempted from participation in PE classes; and (c) not having any diagnosed issue based on review of official medical history. In each school, all students meeting the above-mentioned criteria were included in the study. The total sample was composed of 188 students (88 girls, 100 boys) with an average age of 10.22 ± 0.76 years. Detailed information on the characteristics of the participants in the EG and CG group are presented in Table 1.

### 2.3. Instruments

Physical fitness components were assessed using the Eurofit test battery [37]. The following tests were selected for the purpose of the present research: (a) The Sit-and-Reach test for the assessment of lower-back flexibility. As suggested in the Eurofit manual, the measure at feet level is set at 15 cm. Values may be negative (i.e., students are 16 cm, or more, behind the line of their feet as they bend forward), at “0” (students’ hands are 15 cm behind the line of their feet as they bend forward) or positive; (b) the 20 m endurance shuttle-run test for assessing cardiorespiratory fitness. The reported measure was the total distance covered in meters; (c) the handgrip strength test by means of a Smedley III Dynamometer from Takei, measures being given in kilograms and grams; (d) the 30-s sit-up test for muscular endurance, by which children perform as many repetitions of sit-up abdominals as possible in 30 s; (e) the 10 × 5 m shuttle-run test, evaluating running speed by measuring the total time (in seconds) needed to perform 10 sprints between two cones placed five meters apart; and (f) the standing broad jump, which assesses lower-limb muscular power. The test is performed with a take-off with both feet; arm swinging and knee bending are allowed before the jump. The landing must be on both feet and it is considered valid only if participants do not lose balance or fall afterwards. The total distance covered (centimeters) from the starting line to the point of landing of the heel is measured.

### 2.4. Intervention Program

The intervention lasted six months and was applied during regular PE classes, which in the context of the research have a frequency of twice per week and a duration of 45 min per session. Both EG and CG groups performed three sports evenly distributed in three two-month units. The sports disciplines of football, basketball, and handball were chosen as all schools involved in the present research had sufficient equipment and facilities to practice them properly. Additionally, these sports are commonly included in the PE curriculum taught at this stage of education. During each sub-unit (sport discipline), students were initially presented the discipline; the teachers discussed with them about the characteristics of the given sport, allowing the students to share their previous experiences and express their knowledge and opinion on it (TGfU phase a). The main block of activities consisted in working on different tactical situations, starting from simple structures (1 vs. 1, 1 vs. 2, 2 vs. 1) to the most complex ones (depending on the discipline; for instance, 9 vs. 10 or more in football). A set of challenges was set for the proposed structures. For instance, for attackers, these challenges included reaching a certain area while avoiding defenders’ interference; completing a certain number of passes without being intercepted; or scoring; whilst those students assigned to defense were challenged to find strategies to efficiently block the attackers. At this stage of the program, students had the opportunity to practice independently in small groups each tactical situation introduced (TGfU phase b). After that, teachers and students engaged in debates on what should be done/avoided to succeed in the given situation (TGfU phase c), autonomously trained the necessary skills, and then tried to make use of them in practice (TGfU phases d and e). In TGfU, the teachers may decide to repeat phases c, d, and e as many times as needed, depending on the students’ rate of success and understanding. Every time a debate (phase *c*) is prompted, the discussion is enriched by the students’ experiences from performing the activities (phase d), and skill training (phase e) is modified to adjust to the outcomes of each new discussion. Finally, the last part of the unit (two sessions) was used for the students to experience the full game. The participants in the CG performed traditional PE sports activities as established in the formal national curriculum. This consists of an initial work focused on familiarization with the discipline (rules, spaces, equipment), followed by technical drills based on imitation, repetition, and small-group activities. Successively, tactical aspects are introduced through pre-established game situations, in which the students are previously instructed by the teacher on how to perform them. In the final part of the unit, students play regular games/matches of the standard, full discipline. A typical progression of the activities of both groups is shown in Table 2 as follows.

### 2.5. Procedure

This research was approved by the National Council of Science and Technology (reference number: C00/425/13). All participants had their parents or legal guardians formally consent their participation by signing a consent form. During the semester before the application, the PE teachers teaching at 5th and 6th grade in the schools assigned to the experimental treatment were introduced and trained on the TGfU approach by means of theoretical and practical workshops carried out at the research facilities with the support of bachelor and master students from the research institution. Physical fitness measurement sessions were carried out before and after the intervention period, during the regular schedule of the PE classes, with the permission and assistance of the PE teachers involved. The assessment was performed in three separate sessions both at pre- and post-test. In the first session, flexibility, speed, and handgrip strength tests were carried out. During the second session, participants performed the tests for muscular endurance and power. The final session was exclusively used for the endurance shuttle test.

### 2.6. Statistical Analysis

An initial descriptive and frequency analysis was run for each variable to detect potential input errors or missing values. Additionally, the methods of standardized z scores and Mahalanobis D^2^ were employed to examine the presence of outliers. Students’ t-tests for paired samples were performed to evaluate intra-group differences between pre- and post-test. The effect size was calculated by means of Cohen’s d, using the conventions small (0.2), medium (0.5), and large (0.8), as suggested by Cohen [38]. Inter-group differences at post-test were assessed by means of Analyses of Covariance (ANCOVAs), run per each dependent variable separately, using the outcomes from the pre-test as the covariates. Given that the allocation of participants in the EG or CG was not randomized, introducing pre-test scores as covariates was a necessary step to ensure the meaningfulness of the findings. The effect size for the ANCOVAs was calculated by means of partial eta squared, with the conventions small (0.01), medium (0.06), and large (0.14) [39]. ANCOVAs were also used to check for differences by gender, which is known to be a potential factor in physical fitness.

## 3. Results

Participants in the EG showed significant differences in all components of physical fitness at post-test. Cohen’s d values were 0.63 or higher, indicating a medium to large effect size.

Significant differences were also found for most of the components of physical fitness in the CG, as well. Only muscular endurance, measured with the 30-s abdominal test, showed no statistically significant changes between pre- and post-test (*p* = 0.775). Flexibility, cardiorespiratory fitness, handgrip strength, and low-limb power increased significantly after the six-month period of the research (effect sizes ≥ 0.49); whilst speed decreased significantly at post-test (*p* = 0.001; d > 1). Table 3 shows the pre-post comparison for the EG and CG participants.

Regarding the inter-group analyses at post-test, the initial analyses carried out to verify potential gender differences showed that boys and girls from both EG and CG had similar developmental slopes from pre- to post-test (EG: Handgrip, *p* = 0.53; flexibility, *p* = 0.861; abdominals, *p* = 0.906; low-limb power, *p* = 0.079; CF, *p* = 0.377; and speed, *p* = 0.064; CG: Handgrip, *p* = 0.230; handgrip, *p* = 0.109; abdominals, *p* = 0.146; low-limb power, *p* = 0.338; CF, *p* = 0.115; and speed, *p* = 0.155). Analyzing by treatment, the ANCOVAs showed significant differences in all components of physical fitness; EG participants obtaining higher scores than those in the CG. While these differences were supported by low effect sizes in cardiorespiratory fitness and low-limb power (η = 0.021 and 0.025, respectively), the effect size for the remaining components was from medium to large. The findings from the ANCOVAs are displayed in Table 4.

## 4. Discussion

The objective of this study was to assess the effect of a TGfU-based intervention on physical fitness. To date, the authors have little knowledge of any research carried out to specifically assess whether TGfU modifies the different components of fitness, and in which manner. Despite some authors claiming that the model may be effective for the development of certain fitness domains, the data supporting this suggestion is little. Mandigo et al. [8] implemented an after-school intramural program based on TGfU by adapting it to be consistent with the development of physical literacy in youth. The findings of their research seem to suggest that cardiovascular endurance can be significantly developed through the model within an eight-week period. Nonetheless, these results should be taken with caution, as the authors do not fully adhere to the TGfU model. Instead, they modify it to fit their intervention protocol. Moreover, they do not use this approach within PE, but they propose it as an after-school addition to the already existing PE curriculum. Therefore, the increase in certain variables may have also been partly due to the additional active time the participants were provided with throughout the intervention. Cardiorespiratory benefits of TGfU are proposed also by Morra and Hansen [40], who measured heart rate levels of students during TGfU units, finding that they were at or above their target heart rate zone for longer time than with traditional sports approaches in school settings. In general, our study seems to support the results reported by these authors regarding cardiorespiratory fitness, as our findings suggest a significant increase in endurance accompanied with a large effect size.

However, differently from the previous literature, our work also investigated other areas of fitness that had not yet received full consideration in this research field, i.e., speed, flexibility, and strength. In the past, these components of fitness have been associated with more traditional in-school sports and exercise training forms, mostly based on series and repetitions, or stricter and less game-focused methodological approaches [41,42]. In other cases, sports games were implemented as extra training sessions to be added to the regular school PE [43,44]. Our findings suggest that a student-centered active model, such as TGfU, although including phases focused on discussions and individual/group reflections rather than concretely being in movement, may have a significant effect on these components of physical fitness. Even more, in our sample, the EG participants obtained better scores than the CG ones in each and every fitness component, suggesting that TGfU could represent a more efficient pedagogical approach for the increase of the overall physical fitness in school settings than the traditional sports training methodologies.

The differences between EG and CG in our study may also be partially explained as the result of the mediation of other variables that TGfU seems to have a positive impact on. In fact, previous literature has pointed out the influence that the model has on psychological variables related to youth’s choice of an active lifestyle. Morales-Belando et al. [26] suggested that students involved in TGfU units perceive being active as more enjoyable than their peers participating in traditional PE units. Additionally, a link between participating in TGfU practices and increased motivation towards PA has been shown in different studies [23,26]. Furthermore, TGfU has been widely linked with positive effects on perceived competence [23] and moderate-to-vigorous PA (MVPA) levels [29]. The mentioned variables are known to moderate and directly or indirectly trigger PA habits and higher participation in PA in and out of school. For instance, Eberline, Judge, Walsh, and Hensley [45] found a high effect of enjoyment on children’s engagement and maintenance of active habits. Moreover, motivation towards exercising and increased PA time have been linked in previous studies [46]. Increased PA time seems to be triggered by children’s perception of their skills and motor competence as well, even in the case that perceived competence does not match the real one [47]. Time spent exercising [48], higher engagement in physical activities [49], and MVPA [50] are associated with improved fitness in youth populations. Although we have not directly observed such variables, it is possible that over the implementation period they increased in the students in the TGfU group, and, as a consequence, they may have fostered more active behaviors not only during school time, but also after school.

A final consideration can be pointed out: Some authors suggest that a comprehensive work on different physical fitness components simultaneously may be more effective than training each of them separately [51]. Although the interaction among components has been reported to have small effects, this should be still considered as a potential factor augmenting the magnitude of our outcomes.

## 5. Limitations

The quasi-experimental design does not allow for as robust conclusions as for those studies applying randomized distribution of the participants within the treatment groups. While using quasi-experimental designs, we need to consider the higher risk of selection and maturation biases [52]. Nonetheless, quasi-experimental approaches present several advantages in the field of study of social sciences. In this sense, one of the major benefits in this field is the fact that quasi experiments allow for evaluating real-world effectiveness of a certain treatment, which means that they might be more generalizable and have higher external validity than randomized trials [52]. In studies carried out in schools, especially when innovative pedagogical models are tested, understanding how such models work in real life is essential for the impact they may have on the community.

Especially in school-based programs implemented through PE, teachers’ attitudes and competences play a key role [53]. In our study, experimental and control groups were established through school conglomerates, meaning that some PE teachers exclusively implemented the TGfU program, whereas others only adhered to the traditional approach. Perhaps, in the future, the selection of experimental and control classes may be performed differently so that each PE teacher involved works with both treatments. Nonetheless, this could also increase the risk of certain experimental biases.

Several psychological variables linked to PA habits, levels, and intensity have not been observed in the study. As already discussed, authors have highlighted the link between TGfU and such variables, which, on the other hand, can moderate an increase in PA behaviors, and consequently, in physical fitness. Further research should include the observation of these variables to delve into the connection between TGfU and variations in physical fitness. Perhaps, the assessment of complex structural models comprising the direct/indirect interaction of TGfU with physical fitness along with the contribution and moderation effects of psychological changes (for instance, students’ motivation and enjoyment towards physical activity, or perceived self-competence) could serve as the successive step in this field of research. Such structural models could be complemented by process evaluation strategies assessed with mixed method approaches. This could provide researchers and practitioners with a wider understanding of TGfU effectiveness in different communities, any potential barriers, and other contextual factors that may impact its implementation. Additionally, it would be interesting to monitor students’ changes in their out-of-school activities during the period of implementation. In this way, we could obtain a more precise view of the direct and indirect interconnection between TGfU, psychological variables, PA habits, and physical fitness.

## 6. Conclusions

The present work explores an area of application of the student-centered TGfU model that has not been analyzed in depth to date. Our findings suggest that, beyond the known positive effects of this model on the cognitive and psychological domains, different physical fitness components increase significantly more through TGfU units than through traditional approaches of school sports. This may open up to the use of this model in more PE situations than it commonly is, bringing benefits to students not only in the areas widely discussed in previous research and practice, but also in overall physical health, and more stimulating pedagogical environments in PE. Indeed, as confirmed by Tilga, Hein, Koka, Hamilton, and Hagger [54], controlling and teacher-centered approaches—commonly associated with traditional training methods in and out of school—may lead to a lower perceived quality of life and frustration of basic psychological needs, in contrast with game-based, active approaches, which encourage significant learning and youth comprehensive development.

## Figures and Tables

**Table 1:** Descriptive characteristics of the participants.

Protocol	Gender	*n*	Age		Height (cm)		Weight (kg)	
			Mean	SD	Mean	SD	Mean	SD
Experimental	M	58	10.30	0.81	137.49	5.57	49.77	5.81
	F	47	10.31	0.75	139.28	6.88	49.60	8.03
	Total	105	10.30	0.77	138.48	6.36	49.68	7.09
Control	M	53	10.15	0.72	139.40	8.18	47.12	8.87
	F	30	10.07	0.74	140.90	6.76	48.56	6.06
	Total	83	10.12	0.72	139.94	7.69	47.64	7.96

Note: M: Male; F: Female; SD: Standard Deviation.

**Table 2:** Progression of activities per each sport practiced.

Group	Sport	Sessions 1–4	Sessions 5–8	Sessions 9–12	Sessions 13–16
EG	F	From 1v1 to 3v3	From 3v4 to 6v6	From 6v7 to 9v9	From 9v10 to full game
	B	1v1, 2v1, 1v2	2v2, 3v2, 2v3	3v3, 3v4, 4v3	From 4v4 to full game
	H	From 1v1, 2v1, 2v2	From 2v3 to 4v4	From 4v5 to 6v6	From 6v7 to full game
CG	F, B, H	Basic technique drills	Combined technique drills	Technique execution in fixed situations	Technique execution in fixed situations, full game

Note: EG: Experimental group; CG: Control group; F: Football; B: Basketball; H: Handball.

**Table 3:** Results of the paired pre-post-test comparison for the experimental (EG) and the control group (CG).

Group	Variable	Pre-Test (Mean ± SD)	Post-Test (Mean ± SD)	*p*	d
EG	Flexibility (cm)	4.90 ± 10.65	10.18 ± 8.38	<0.001	0.81
	Abdominals (n)	13.00 ± 4.91	16.54 ± 4.91	<0.001	>1
	Handgrip (kg)	16.36 ± 4.23	17.88 ± 4.07	<0.001	0.88
	CF (m)	338.10 ± 184.59	361.62 ± 178.39	<0.001	0.73
	Low-limb power (cm)	114.93 ± 20.76	120.94 ± 19.88	<0.001	>1
	Speed (seconds)	22.31 ± 2.84	21.89 ± 2.78	<0.001	0.63
CG	Flexibility (cm)	−3.48 ± 17.19	−0.61 ± 15.55	<0.001	0.63
	Abdominals (n)	16.67 ± 4.42	16.61 ± 4.26	0.775	
	Handgrip (kg)	18.11 ± 4.46	18.73 ± 4.48	<0.001	>1
	CF (m)	267.71 ± 109.49	284.82 ± 110.19	<0.001	0.49
	Low-limb power (cm)	133.13 ± 21.99	136.00 ± 20.76	<0.001	0.59
	Speed (seconds)	21.99 ± 2.15	22.45 ± 2.16	0.001	>1

Note: SD: Standard Deviation; CF: Cardiorespiratory fitness.

**Table 4:** Results of the univariate analysis of covariance for the analysis of inter-group differences at post-test.

Variable	EG (EMM ± SD)	CG (EMM ± SD)	F	*p*	η
Flexibility (cm)	7.26 ± 0.49	3.09 ± 0.56	29.780	<0.001	0.139
Abdominals (n)	17.96 ± 0.21	14.82 ± 0.24	92.166	<0.001	0.333
Handgrip (kg)	18.58 ± 0.16	17.85 ± 0.17	9.749	0.002	0.050
CF (m)	332.01 ± 3.21	322.28 ± 3.62	3.953	0.048	0.021
Low-limb power (cm)	128.35 ± 0.51	126.63 ± 0.57	4.691	0.032	0.025
Speed (s)	21.76 ± 0.09	22.62 ± 0.10	37.942	<0.001	0.170

Note: EG: Experimental Group; CG: Control Group; EMM: Estimated Marginal Means; SD: Standard Deviation; CF: Cardiorespiratory fitness.
